# Unraveling the Origin of Exceptional Activity in NiMo Alloys for Alkaline Hydrogen Evolution

**DOI:** 10.1002/advs.202518742

**Published:** 2025-12-27

**Authors:** Yuefeng Zhang, Wenqiang Yang, Zhiyuan Zeng, Zhen‐Yu Wu, Zhenbin Wang

**Affiliations:** ^1^ Department of Materials Science and Engineering City University of Hong Kong Hong Kong SAR China; ^2^ Department of Chemistry Institute of Innovative Material Guangdong Provincial Key Laboratory of Sustainable Biomimetic Materials and Green Energy Southern University of Science and Technology Shenzhen China; ^3^ Department of Chemical Engineering University of South Carolina Columbia South Carolina USA; ^4^ School of Energy and Environment City University of Hong Kong Hong Kong SAR China

**Keywords:** hydrogen evolution reaction, microkinetic modeling, NiMo alloy, operando‐stable surface

## Abstract

Nickel‐molybdenum (NiMo) alloys show benchmark alkaline hydrogen evolution reaction (HER) activity, yet the active phase and mechanism remain debated. Here, we resolve this ambiguity by combining density functional theory with surface Pourbaix diagrams to model the catalyst under realistic operando conditions. We find that catalyst surfaces are reconstructed by oxygen and that a clear synergistic mechanism emerges: Mo sites catalyze the rate‑determining Volmer step (water dissociation), while adjacent Ni sites provide near‐optimal binding for hydrogen evolution. This synergy is most pronounced on the O‐covered Ni_3_Mo(111) facet, which exhibits a low water dissociation barrier (Δ*G*_a_ = 0.65 eV) and near‐thermoneutral hydrogen adsorption (Δ*G*_H_ = −0.01 eV), explaining its superior performance. Furthermore, our microkinetic model quantitatively validates this mechanism by predicting an exchange current density in excellent agreement with experimental values. Our findings also challenge the recent assignment of MoO_x_ as the active site. This work establishes a definitive mechanistic framework that reconciles prior controversies and provides rational design principles for HER catalysts.

## Graphical abstract

This study resolves the controversy regarding the active origin of Nickel‐molybdenum (NiMo) alloys for alkaline hydrogen evolution. By combining surface Pourbaix diagrams with microkinetic modeling, we demonstrate that the O‐covered Ni_3_Mo surface drives activity through a synergistic mechanism: Mo sites facilitate water dissociation while adjacent Ni sites optimize hydrogen evolution, definitively ruling out MoO_x_ as active species.

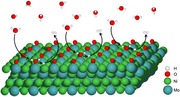

## 1 Introduction

Green hydrogen production via water electrolysis is vital for a sustainable energy future. Anion Exchange Membrane Water Electrolysis (AEMWE) stands out in this context due to its use of affordable, non‐precious metal catalysts. However, the slow kinetics of the hydrogen evolution reaction (HER) under alkaline conditions remain a major barrier, driving the search for more efficient catalysts. Nickel‐molybdenum (NiMo) alloys‐the leading catalysts for alkaline HER‐were first studied extensively between the 1980s and 1990s [1, 2] and have recently regained attention as promising candidates. Current studies primarily focus on optimizing their performance through nanostructuring and morphology engineering [3, 4], leading to breakthroughs such as nanoporous NiMo alloys [3] that achieve high catalytic activity with overpotentials as low as 10∼15 mV at 10 mA/cm^2^.

Despite their record‐breaking HER activity, a consensus on the underlying mechanism for NiMo alloys remains elusive and controversial. Several conflicting theories have been proposed to explain their high performance. One prevailing theory [5, 6] suggests that Ni sites act as primary centers for H_2_O adsorption and dissociation, after which the resulting hydrogen intermediates (H^*^) either form H_2_ directly on the Ni or migrate to adjacent Mo sites for enhanced H_2_ generation. An alternative model [7] proposes the opposite, with H_2_O dissociation occurring at Mo sites and subsequent H_2_ formation on Ni or Mo. A third perspective [8] emphasizes a synergistic mechanism where both Ni and Mo sites work in concert to stabilize the H^*^ intermediate. Finally, a fourth hypothesis involves surface reconstruction, [9] where Mo migrates to the surface and reacts with oxygen‐containing species from air or solution to form MoO_x_/Ni(OH)_2_ species with an expanded specific surface area, thereby facilitating H_2_O adsorption. These conflicting mechanisms highlight a fundamental gap in our understanding of the true origin of NiMo's exceptional catalytic activity.

To resolve these conflicting theories, we combine density functional theory (DFT) with surface Pourbaix diagrams to model the catalyst surface under realistic alkaline HER conditions. Our analysis reveals that oxygen reconstruction fundamentally alters the catalytic landscape, with the O‐covered Ni_3_Mo(111) facet emerging as the primary active phase. Through systematic thermodynamic and kinetic analysis, we demonstrate a synergistic mechanism where Mo and Ni sites perform complementary functions—addressing the rate‐limiting water dissociation and hydrogen evolution steps, respectively. This mechanism is quantitatively validated through microkinetic modeling. These findings not only challenge the recent assignment of MoO_x_ as the active site but also establish design principles for developing next‐generation alkaline HER catalysts.

## 2 Results

### 2.1 Operando‐Stable Surface

**FIGURE 1:**
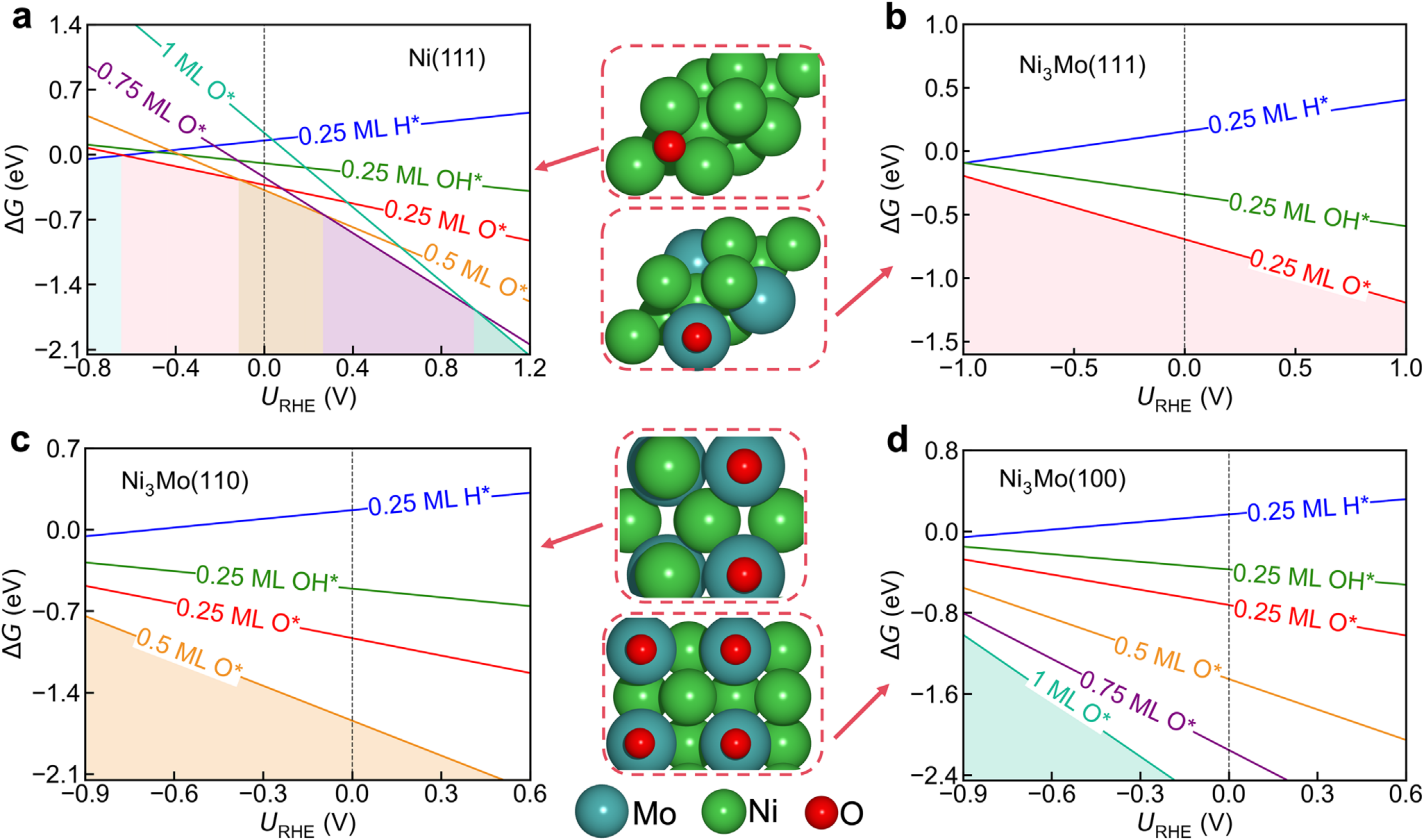
Surface Pourbaix diagrams calculated for alkaline conditions (pH14) for (a) Ni(111), (b) Ni_3_Mo(111), (c) Ni_3_Mo(110), and (d) Ni_3_Mo(100) surfaces. The middle column shows top views of the thermodynamically stable surface states under operating conditions.

Determining the surface adsorption state under electrochemical conditions is a critical first step for mechanistic studies because surface coverage strongly influences the computed energetics. To this end, we calculated the surface Pourbaix diagrams for the Ni(111) and Ni_3_Mo systems (Figure 1). For the Ni(111) surface, H^*^ coverage is favored at potentials below −0.65 V vs. RHE. As the potential increases, H^*^ adsorption weakens, while O^*^ and OH^*^ adsorption become more favorable, with O^*^ binding more strongly than OH^*^. Consequently, the surface is covered by 0.25 monolayer (ML) of O^*^ in the potential window of −0.65 to −0.13 V, transitioning to 0.5 ML O^*^ from −0.13 to 0.28 V. Given that hydrogen evolution on Ni catalysts typically occurs at potentials more negative than −0.2 V vs. RHE, [10] we identify the 0.25 ML O^*^‐covered surface as the most relevant state under alkaline HER operating conditions.

A similar analysis was performed for the Ni_3_Mo surfaces. Under typical HER working conditions, the stable surfaces were determined to be Ni_3_Mo(111) covered by 0.25 ML O^*^, Ni_3_Mo(110) by 0.5 ML O^*^, and Ni_3_Mo(100) by 1.0 ML O^*^ (Figure 1b‐d). These oxygen coverages correlate directly with the number of Mo atoms on each respective surface, consistent with the higher oxophilicity of Mo compared to Ni [11]. The determined surface states for the Ni(111) and Ni_3_Mo systems under alkaline HER conditions are shown in the middle column of Figure 1. Note that the surface Pourbaix diagrams presented here reflect the surface state at thermodynamic equilibrium. Under dynamic electrochemical conditions, factors such as local pH gradients and scan rates may lead to deviations from these predictions. However, identifying the thermodynamically stable coverage provides a necessary theoretical baseline and indicates that the HER mechanism proceeds on an O‐decorated surface rather than the pristine metallic phase.

### 2.2 Thermodynamic Activity

**FIGURE 2:**
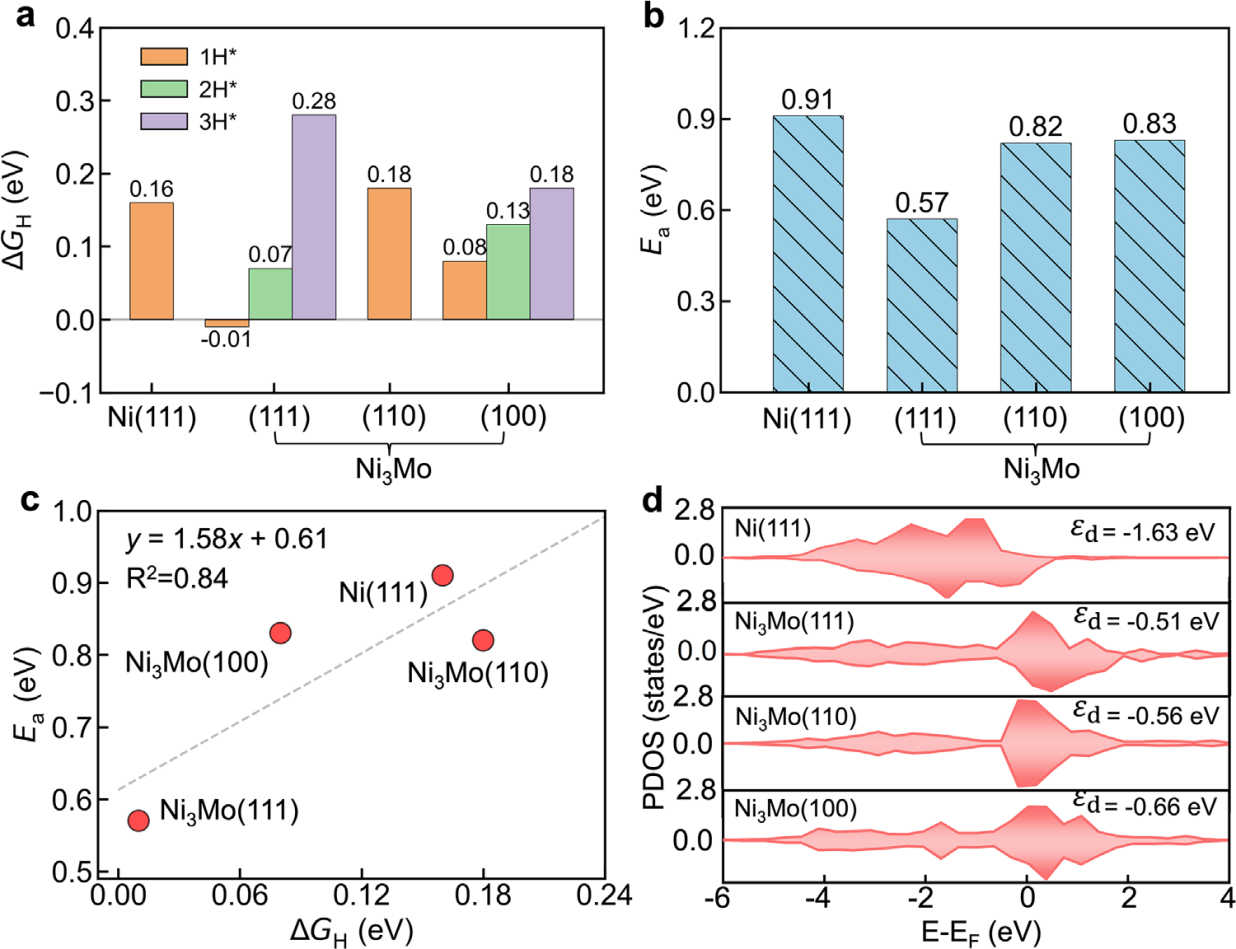
Calculated (a) hydrogen adsorption free energies (Δ*G*_H_) and (b) activation barriers (*E*_a_) for water dissociation on Ni(111), Ni_3_Mo(111), Ni_3_Mo(110), and Ni_3_Mo(100) surfaces. (c) Linear scaling relationship between Δ*G*_H_ and *E*_a_ across these surfaces. (d) Projected *d*‐orbital density of states at the active sites on these catalyst surfaces, with the *d*‐band centers labeled.

The HER activity volcano suggests that an ideal catalyst should exhibit a differential Gibbs free energy for hydrogen adsorption (Δ*G*_H_) close to zero. [12] Accordingly, we calculated Δ*G*_H_ for Ni(111) and three Ni_3_Mo surfaces based on their surface states under alkaline conditions (Figure 2a). The structures of the most active H^*^ adsorption sites are provided in Figure S2. Hydrogen adsorption on the Ni(111) surface is thermodynamically uphill, with a Δ*G*_H_ of 0.16 eV, indicating weak hydrogen binding. In contrast, the first hydrogen adsorption step on Ni_3_Mo(111) is downhill, with a near‐optimal Δ*G*_H_ of −0.01 eV. The second and third hydrogen adsorption steps on Ni_3_Mo(111) become progressively less favorable, with Δ*G*_H_ of 0.07 and 0.28 eV, respectively. These results suggest that the first hydrogen adsorption step is primarily responsible for HER activity, as its Δ*G*_H_ is closest to zero. The calculated Δ*G*_H_ values for Ni_3_Mo (110) and Ni_3_Mo(100) are 0.18 and 0.08 eV, respectively. These results indicate that the HER activity among the Ni_3_Mo surfaces follows the trend: Ni_3_Mo(111) > Ni_3_Mo(100) > Ni_3_Mo(110). Therefore, Ni_3_Mo(111) is likely the dominant facet responsible for the experimentally observed high HER activity in alkaline solutions.

For comparison, we also evaluated Δ*G*_H_ for Ni(111) and the three Ni_3_Mo surfaces under acidic conditions (Figure S2a–d). The Ni_3_Mo surfaces show Δ*G*_H_ values close to zero: −0.03 eV for Ni_3_Mo(111), −0.06 eV for Ni_3_Mo(110), and 0.04 eV for Ni_3_Mo(100), compared to −0.18 eV for Ni(111). The corresponding most active configurations are shown in the middle column of Figure S3. These results suggest that the Ni_3_Mo alloy is also highly active for HER in acidic solutions, consistent with experimental observations [13]. The activity trend in acidic media is Ni_3_Mo(111) ≈ Ni_3_Mo(110) ≈ Ni_3_Mo(100) > Ni(111), which differs from that observed under alkaline conditions. While both sets of DFT calculations predict that Ni_3_Mo exhibits high HER activity, they reveal nuanced differences in underlying mechanisms at the atomistic level.

### 2.3 Kinetic Activity

In alkaline solutions, water is the proton source for hydrogen evolution, and its dissociation (Volmer step) is regarded as the rate‐determining step. [14, 15] We therefore calculated the water dissociation barrier on Ni(111) and three Ni_3_Mo surfaces (Figure 2b). Water dissociation on Ni(111) has a calculated activation barrier (*E*_a_) of 0.91 eV and an elongated O─H bond length of 1.55 Å at the transition state (TS) (Figure S4a). In contrast, the water dissociation barrier on Ni_3_Mo(111) is significantly reduced to 0.57 eV, indicating faster reaction kinetics compared with Ni(111). A detailed analysis reveals that water dissociation on Ni_3_Mo(111) occurs on the Mo site, with an O─H bond length of 1.35 Å at the TS (Figure S4b). The resulting OH^*^ species occupies the Mo site, while the H^*^ species is directly transferred to the adjacent Ni site simultaneously. Furthermore, the calculated water dissociation barriers on Ni_3_Mo(110) and Ni_3_Mo(100) are 0.82 and 0.83 eV, respectively, with corresponding O─H bond lengths at the TS of 1.33 and 1.36 Å (Figure S4c,d). These results indicate that water dissociation is more kinetically favorable on all Ni_3_Mo surfaces compared to Ni(111). The trend of kinetic activity for HER thus follows the order: Ni_3_Mo(111) > Ni_3_Mo(110) ≈ Ni_3_Mo(100) > Ni(111). Additionally, we find a strong linear scaling relationship between Δ*G*_H_ and *E*_a_, with a correlation coefficient of 0.84 (Figure 2c). Notably, including HER activity for MoO_2_ (vide infra) and MoO_2_(OH)_2_ cluster improves the linear correlation coefficient to 0.97 (Figure S5). This linear relationship holds true at different surface coverages and applied potentials (Figure S6). These results confirm that Δ*G*_H_ is a good thermodynamic HER activity descriptor from a kinetic perspective.

To understand the origin of the fast reaction kinetics of NiMo catalysts, we further calculated the d‐band center (ε_*d*_) of the active Ni and Mo sites responsible for HER on each surface (Figure 2d). An ε_*d*_ closer to the Fermi level corresponds to a stronger interaction between the metal surface and the adsorbate. [16] The ε_*d*_ values of the Ni atom on Ni(111) and Mo atom on Ni_3_Mo(111), Ni_3_Mo(110) and Ni_3_Mo(100) are −1.63, −0.51, −0.56, and −0.66 eV, respectively, indicating that Ni(111) have the weakest binding for TS, resulting in the highest water dissociation barrier and thereby explaining its relatively poor HER activity. In contrast, the active Mo site on Ni_3_Mo(111) exhibits the strongest binding and thereby effectively stabilizes the TS, resulting in the lowest kinetic barrier for hydrogen evolution. These results rationalize the predicted activation energies for the rate‐determining‐step of HER on Ni(111) and Ni_3_Mo catalysts. Additionally, the higher ε_*d*_ value of Ni_3_Mo(111) compared to Ni_3_Mo(110) and Ni_3_Mo(100) clarifies the fundamental reason for the varied water dissociation barriers on Ni_3_Mo surfaces despite their similar TS O─H bond lengths (Figure S4b‐d).

### 2.4 Solvation and Potential Effects

**FIGURE 3:**
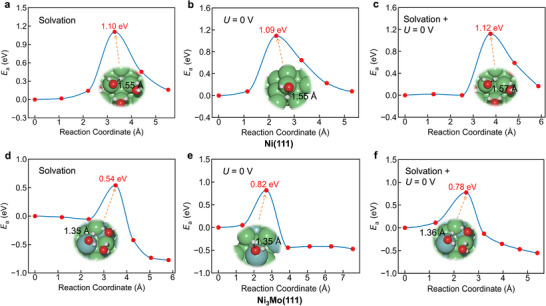
Calculated kinetic energy profiles for water dissociation on Ni(111) (a–c) and Ni_3_Mo(111) (d–f) under three conditions: (a,d) with solvation only (modeled explicitly and implicitly), (b,e) at a constant potential of 0 V vs. SHE, and (c,f) with both effects combined. Here, 0 V vs. SHE corresponds to −4.44 V on the absolute scale.

It is widely acknowledged that solvation and electrode potential play important roles in electrochemical reactions, whereas standard DFT typically models reactions in vacuum under constant‐charge conditions. Therefore, we investigated the effects of solvation and potential on the water dissociation barriers on Ni(111) and Ni_3_Mo(111) surfaces. For Ni(111) (Figure 3a‐c), the calculated *E*_a_ values with solvation only, at a constant potential of 0 V vs. SHE, and with both effects combined are 1.10, 1.09, and 1.12 eV, respectively. Compared to the standard DFT barrier of 0.91 eV (Figure 2b), each treatment increases *E*_a_ by about 0.2 eV. These results suggest not only that water dissociation is more challenging under realistic alkaline HER conditions, but also that the effects of solvation and potential on the DFT‐predicted barrier are minimal for Ni(111). This qualitatively confirms that Ni metal is not a good alkaline HER catalyst and typically requires large overpotentials to achieve decent current density in practical applications [1, 2].

For Ni_3_Mo(111) (Figure 3d‐f), the *E*_a_ values with solvation only, at a constant potential of 0 V vs. SHE, and with both effects combined are 0.54, 0.82, and 0.78 eV, respectively. Compared to the standard DFT barrier of 0.57 eV (Figure 2b), solvation has a minimal effect (−0.03 eV), whereas applying a constant potential increases *E*_a_ by 0.25 eV; including both effects yields a 0.21 eV increase. These results indicate that the effect of potential plays a larger role than solvation in determining the HER barrier on Ni_3_Mo(111). Notably, the activation free energy (*G*_a_) on Ni_3_Mo(111) is 0.65 eV under solvation at *U* = 0 V (Figure S7), which further suggests that water splitting on Ni_3_Mo(111) is experimentally feasible. Across all treatments, *E*_a_ on Ni_3_Mo(111) is consistently lower than on Ni(111) by at least 0.27 eV, indicating substantially faster HER kinetics. This result is in excellent agreement with experimental findings that Ni_3_Mo catalysts are more active than Ni for HER [17].

To quantitatively link our atomistic simulations with macroscopic experimental observables, we developed a microkinetic model to calculate the exchange current density (*j*_0_), a key metric of intrinsic HER activity. The model predicts a *j*_0_ of 1.53 mA/cm^2^ for the Ni_3_Mo(111), which shows excellent agreement with the experimentally measured value of 1.24 mA/cm^2^. [4] This remarkable consistency provides compelling quantitative support for our theoretical analysis that Mo serves as the active site for the rate‐determining step in the alkaline HER. Additionally, the *j*_0_ calculated using the free energy barrier in vacuum is 7980 mA/cm^2^, which is three orders of magnitude larger than the experimental result. This stark discrepancy highlights that incorporating constant potential and solvation effects is indispensable for accurately modeling the electrochemical interface and achieving quantitative agreement with experimental results.

In addition, calculations under varying constant potentials show that *E*_a_ decreases with increasing overpotential toward HER (Figure S8). This trend is consistent with previous grand‐canonical DFT studies on Pt for HER [18] and with experimental observations that increasing the applied potential enhances HER activity (i.e., lowers *E*_a_) and increases current density.

## 3 Discussion

**FIGURE 4:**
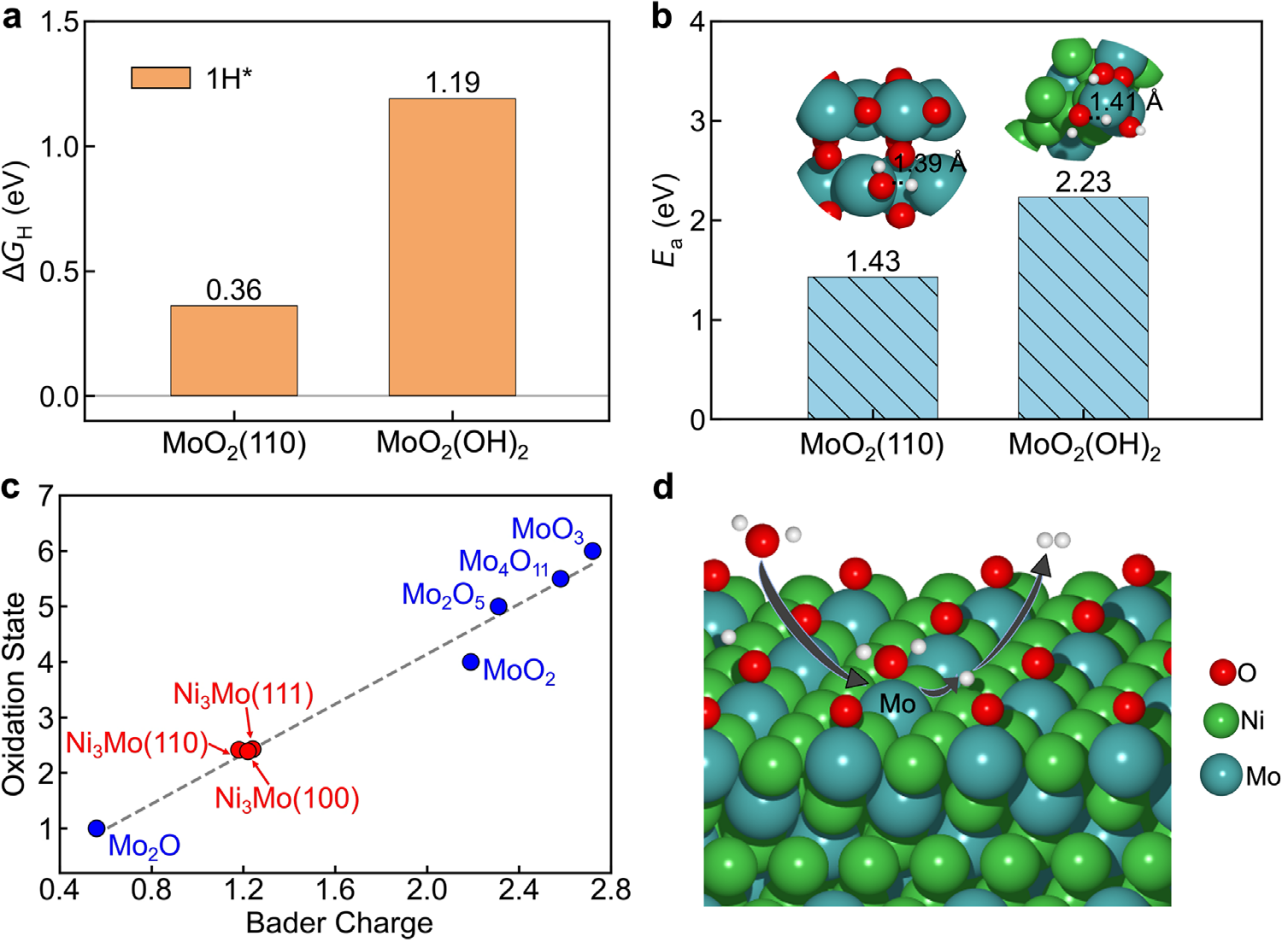
Calculated (a) hydrogen adsorption free energies (Δ*G*_H_) and (b) water‐dissociation barriers (*E*_a_) on MoO_2_(110) and on a MoO_2_(OH)_2_ cluster supported on the Ni_3_Mo(111) surface. The inset presents the corresponding TS configuration. (c) Correlation between Bader charge and oxidation state. (d) Schematic of the synergetic mechanism for hydrogen evolution on NiMo alloys.

While NiMo alloys have long been recognized as the most active catalysts for the alkaline HER, the nature of their active sites and the reaction mechanisms remain under debate. Recent in situ experimental studies [9] have suggested that molybdenum oxides (MoO_x_) are responsible for this high activity. Our calculations, however, challenge this conclusion. On MoO_2_(110), the calculated Δ*G*_H_ and water‐dissociation barrier are 0.36 eV and 1.43 eV, respectively; for a MoO_2_(OH)_2_ cluster on Ni_3_Mo, these values increase to 1.19 and 2.23 eV (Figure 4a,b). Both sets of values are considerably higher than those on Ni_3_Mo(111), indicating poor intrinsic HER activity. To completely rule out MoO_x_ as an active site, we further evaluated oxygen vacancy (V_O_) formation under reducing conditions. The calculated V_O_ formation energy on MoO_2_(110) at an applied potential of −0.5 V vs. RHE is 4.25 eV, indicating that oxygen vacancies cannot form under typical HER operating conditions. Therefore, both thermodynamic and kinetic analysis suggest that MoO_x_ is unlikely to be the active site.

Considering that Ni(OH)_2_ and NiMoO_4_ may coexist with MoO_x_ on the reconstructed NiMo alloy surfaces and interfaces [19, 20, 21, 22, 23], we evaluated the HER activity of these phases (Figure S9). Our calculations demonstrate that both species are intrinsically inactive for HER. This confirms that these reconstructed phases are not responsible for the experimentally observed high HER activity.

Further supporting this, our Bader charge calculations show that Mo atoms on the Ni_3_Mo alloy surfaces exhibit an oxidation state near +3 (Figure 4c), consistent with previous in‐situ measurements [9]. Based on this evidence, we propose a synergistic mechanism where water is activated and dissociated at Mo sites, and the resulting hydrogen evolves at adjacent Ni sites (Figure 4d). This model readily explains experimental observations where leaching of Mo atoms from the alloy surface significantly decreases catalytic activity. The dissolution of Mo removes the optimal sites for the rate‐determining Volmer step, thereby shifting the water dissociation process from Mo sites with a low kinetic barrier to Ni sites with a much higher one. Notably, the trend of Mo‐induced activation is further confirmed by the calculated thermodynamic and kinetic activity on NiMo(111) (Figure S10), which exhibits high activity comparable to that of Ni_3_Mo(111). This insight offers a clear design principle for new catalysts: engineering surfaces that balance water activation capabilities with hydrogen binding strength through strategic site pairing.

A key distinction in our work is the methodology used. Previous DFT calculations that interpreted experimental observations were typically performed under vacuum conditions. In contrast, our study predicts HER activity using an operando‐stable surface determined from surface Pourbaix analysis. We contend that to reliably interpret experimental findings, DFT calculations must employ a surface state representative of the electrochemical working conditions. A critical question remains, however: why do in situ experiments detect MoO_x_ species on NiMo alloy surfaces? We propose three potential explanations:

- Air Oxidation During Handling: Molybdenum is strongly oxophilic and oxidizes readily, even in air. The detected MoO_x_ could be an artifact of unintentional oxidation during sample preparation.
- Dissolution and Redeposition: Both theoretical Ni‐Mo Pourbaix analysis [24] and experimental studies [25] have shown that Mo readily dissolves under alkaline conditions to form molybdate (MoO_4_^2−^) species. These species could then form MoO_2_(OH)_2_ species and dynamically redeposit onto the catalyst surface as MoO_x_.
- Operando surface oxidation: According to our calculated surface Pourbaix diagram (Figure 1), the NiMo alloy surface itself is prone to oxidation under working conditions, which would form Mo oxo‐species.

## 4 Conclusion

In summary, we have resolved the long‐standing controversy over the origin of NiMo alloys’ exceptional alkaline HER activity. By integrating DFT with surface Pourbaix analysis to explicitly account for operando surface states, we demonstrate that the O‐covered Ni_3_Mo(111) facet is the dominant active surface. A clear synergistic mechanism emerges, where Mo sites catalyze the rate‐determining Volmer step (water dissociation) with a moderate barrier (Δ*G*_a_ = 0.65 eV), while adjacent Ni sites provide near‐thermoneutral H binding (Δ*G*_H_ = −0.01 eV) for efficient H_2_ evolution. Crucially, our microkinetic model provides quantitative validation for this mechanism, predicting an exchange current density that is excellent agreement with experimental measurements. This unified thermodynamic‐kinetic picture not only rationalizes the superior performance of Ni_3_Mo(111) over other low‐index facets but also definitively challenges the prevailing assignment of MoO_x_ as the intrinsically active phase. By establishing the critical importance of realistic surface state analysis and elucidating the synergistic roles of Ni and Mo, this work provides a clear mechanistic foundation for the rational design of high‑performance alkaline HER electrocatalysts.

## Conflicts of Interest

The authors declare no conflicts of interest.

## Supporting information




**Supporting File**: advs73558‐sup‐0001‐SuppMat.docx.

## Data Availability

The data that support the findings of this study are available from the corresponding author upon reasonable request.
