# Supplementary material for: Unraveling the Origin of Exceptional Activity in NiMo Alloys for Alkaline Hydrogen Evolution
Source: Adv Sci (Weinh). 2025 Dec 27;13(14):e18742. doi: 10.1002/advs.202518742 (PMC12970241; doi:10.1002/advs.202518742)
Supplement: Supplementary file 1 — Supporting File: advs73558‐sup‐0001‐SuppMat.docx. [file ADVS-13-e18742-s001.docx]

Supplementary Information

Unraveling the Origin of Exceptional Activity in NiMo Alloys for Alkaline Hydrogen Evolution

Yuefeng Zhang^1,2^, Wenqiang Yang^3^, Zhiyuan Zeng^1*^, Zhen-Yu Wu^2*^, Zhenbin Wang^1,4*^

^1^Department of Materials Science and Engineering, City University of Hong Kong, Hong Kong SAR, 999077, China

^2^Department of Chemistry, Institute of Innovative Material, Guangdong Provincial Key Laboratory of Sustainable Biomimetic Materials and Green Energy, Southern University of Science and Technology, Shenzhen 518055, China

^3^Department of Chemical Engineering, University of South Carolina, 301 S. Main Street, Columbia, South Carolina 29208, United States

^4^School of Energy and Environment, City University of Hong Kong, Hong Kong SAR, 999077, China

E-mails: zwan22@cityu.edu.hk; wuzy@sustech.edu.cn; zhiyzeng@cityu.edu.hk

# Computational methods

## Density Functional Theory Calculation

Spin-polarized density functional theory calculations were performed using the Vienna ab initio simulation package within projected-augmented wave method.^[1,2]^ The revised Perdew-Burke-Ernzerhof (RPBE) functional was employed.^[3]^ A plane-wave basis with a kinetic energy cutoff of 400 eV was used. To present interactions between periodic images, a vacuum layer of 16 Å was added along the z-direction. The convergence criteria for the total energy and atomic force were set to 10^−5^ eV and 0.05 eV/Å, respectively. Transition states (TS) and reaction barriers were identified using the climbing-image nudged elastic band (CI-NEB) method.^[4]^ The nature of each TS was verified through vibrational frequency analysis, ensuring the presence of a single imaginary frequency.

For pure nickel, the face-centered cubic Ni(111) facet was modeled, as it is the most stable surface and features four distinct adsorption sites (Figure S1a). For NiMo alloy, we investigated the active Ni_3_Mo composition^[5,6]^ focusing on the (111), (110), and (100) surfaces (Figure S1b-d). All surfaces were constructed as four-layers periodic slabs. A 2 × 2 supercell was used for the Ni(111), Ni_3_Mo(111), Ni_3_Mo(100) surfaces, while a 1 × 2 supercell was employed for Ni_3_Mo(110). During structure optimization, the bottom two layers of each slab were fixed at their bulk positions, while the top two layers and adsorbates were allowed to relax. Brillouin zone sampling was performed using a Gamma-centered scheme with the k-point meshes of 5 × 5 × 1 for Ni(111) and Ni_3_Mo(111), 5 × 3 × 1 for Ni_3_Mo(110), and 3 × 3 × 1 for Ni_3_Mo(100). The atomic simulation environment (ASE) tool was employed to visualize and analyze atomistic simulations.^[7]^ The d-orbital-projected density of states was calculated using GPAW with an energy cutoff of 600 eV for plane‑wave basis set.^[8]^ All other parameters were the same as in the VASP calculations. Explicit solvation was modeled using three-molecule water clusters, while the implicit solvent and constant potential were simulated with the VASPsol++ code.^[9]^ Bader charge method was employed to determine the valence state of Mo, using the code developed by the Henkelman group.^[10]^

## Hydrogen Adsorption Free Energy Calculation

The differential hydrogen adsorption energy ($\Delta E_{H}$) on the Ni-based surfaces was calculated as:

$\Delta E_{H}=E[slab+nH]-E[slab+(n-1)H]-\frac{1}{2} E[H_{2}]$ (1)

where $E[slab+nH]$ and $E[slab+(n-1)H]$ are the total energies of surfaces with *n* and *n-*1 adsorbed hydrogen atoms, respectively. $E\left[ H_{2} \right]$ is the total energy of the gas phase H_2_ molecule in a 15 Å cubic box.

The differential hydrogen adsorption free energy ($\Delta G_{H}$) was then calculated as:

$\Delta G_{H}=\Delta E_{H}+ \Delta E_{\mathrm{ZPE}}-T\Delta S$ (2)

Similarly, the activation free energy barrier ($G_{a}$) is expressed as follows:

$$G_{a}=E_{a}+ \Delta E_{\mathrm{ZPE}}-T\Delta S$$

where, $\Delta E_{\mathrm{ZPE}}$ and $\Delta S$ represent the changes in zero-point energy and entropy, respectively, and T = 298.15 K.

Surface Pourbaix Diagram Calculation

The surface Pourbaix diagram identified the real surface state of catalysts under aqueous solutions as a function of pH and the electrode potential,^[11,12]^ represented as:

$S{-O}_{m}H_{n}^{*} + \left( 2m-n \right)\left( H^{+}+e^{-} \right)\leftrightharpoons S^{*}+mH_{2}O$ (3)

where *m* and *n* denote the number of oxygen and hydrogen atoms in the adsorbates, and S denotes an arbitrary surface. The free-energy change for a given surface state under applied potential and pH is given by:

$\Delta G\left( U, pH \right)= G_{S^{*}}+mG_{H_{2}O}-G_{S-O_{m}H_{n}^{*}}-(2m-n)(G_{e^{-}}+G_{H^{+}})$ (4)

At non-standard conditions, the combined free energy of the proton and electron can be approximated by:

$G_{e^{-}}+G_{H^{+}}$ = ${\frac{1}{2}G}_{H_{2}}-$ e$U_{\mathrm{SHE}}$ $+ k_{B}T$(ln[$a_{H^{+}}$]) (5)

Substituting Eq. (5) into Eq. 4 and using ln[$a_{H^{+}}$] = -2.303 pH, the expression becomes:

$\Delta G\left( U, pH \right)= G_{S^{*}}+mG_{H_{2}O}-G_{S-O_{m}H_{n}^{*}}-(2m-n)(\frac{1}{2}G_{H_{2}}-U_{\mathrm{SHE}}-2.303k_{B}T pH)$ (6)

The potential relative to the standard hydrogen electrode (SHE) relates to that on the reversible hydrogen electrode (RHE) scale by:

$U_{\mathrm{SHE}}= U_{\mathrm{RHE}}- k_{B}Tln(10)pH$ (7)

Substituting into Eq. (6), the final form of the free energy change, referenced to the RHE scale is:

$\Delta G\left( U, pH \right)= G_{S^{*}}+mG_{H_{2}O}-G_{S-O_{m}H_{n}^{*}}-(2m-n)(\frac{1}{2}G_{H_{2}}-U_{\mathrm{RHE}})$ (8)

In this work, the pH was set to 14 to simulate the alkaline environment in which the catalyst operated.

Microkinetic Modeling

The microkinetic model was built upon that water dissociation functions as the rate-determining step, with all other elementary steps assumed to be in quasi-equilibrium. Under alkaline conditions, protons are supplied by this water dissociation step, described by the following reaction:

${H_{2}O}_{\mathrm{top}}^{\#}+ *_{\mathrm{fcc}} \frac{\frac{k_{f}}{\rightleftharpoons}}{k_{b}} \mathrm{OH}_{\mathrm{top}}^{\#}+ H_{\mathrm{fcc}}^{*}$ (9)

here, # denotes a Mo site, * denotes a Ni site, and $*_{\mathrm{fcc}}$ refers to a free fcc binding site on catalyst surface. The forward (*k*_f_) and backward (*k*_b_) rate constants are given by:

*k*_f_ = A $\times$exp($\frac{-\Delta G_{f}}{k_{B}T}$) (10)

*k*_b_ = A $\times$exp($\frac{-\Delta G_{b}}{k_{B}T}$) (11)

where the pre-exponential factor is A$\approx{10}^{13} s^{-1}$. The terms $\Delta G_{f}$ and $\Delta G_{b}$ are the activation free energies for the forward and backward reactions, respectively. The overall reaction rate ($\gamma$) and corresponding current density (*j*) are calculated as

$\gamma=$ k_f_ $\times\theta_{H_{2}O}^{\mathrm{top}} \times(1-\theta_{H}^{\mathrm{fcc}})$ $-$ *k*_b_ $\times\theta_{\mathrm{OH}}^{\mathrm{top}} \times\theta_{H}^{\mathrm{fcc}}$ (12)

*j* = n_e_ $\times$ e $\times$ C_s_ $\times$ $\gamma$ (13)

where *n*_e_ denotes the number of electrons transferred, *e* is the elementary charge, and *C*_s_ is the site density.


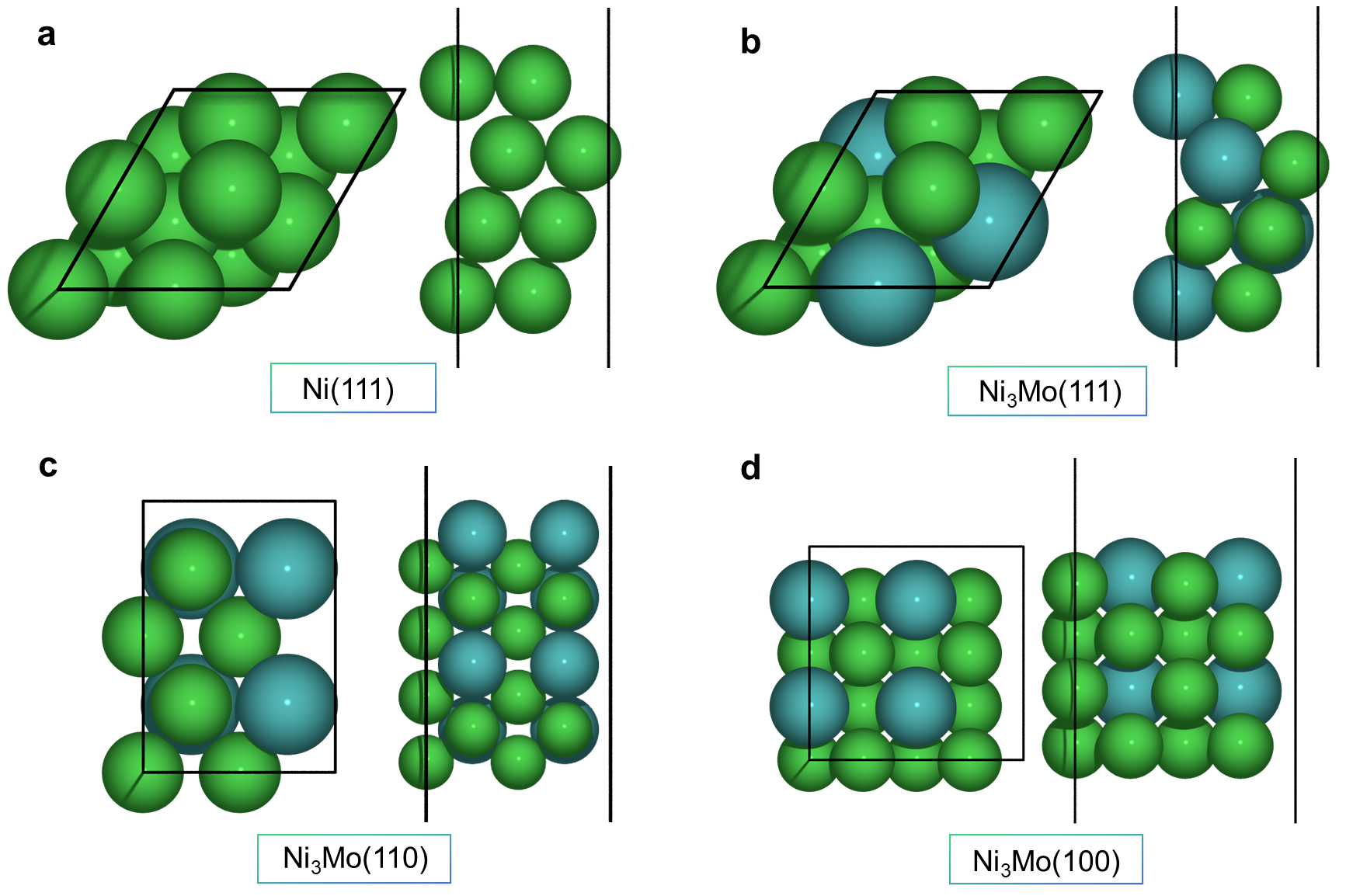


**Figure S1**. Top and side views of the surface models for (a) Ni(111), (b) Ni_3_Mo(111), (c) Ni_3_Mo(110), and (d) Ni_3_Mo(100), respectively.


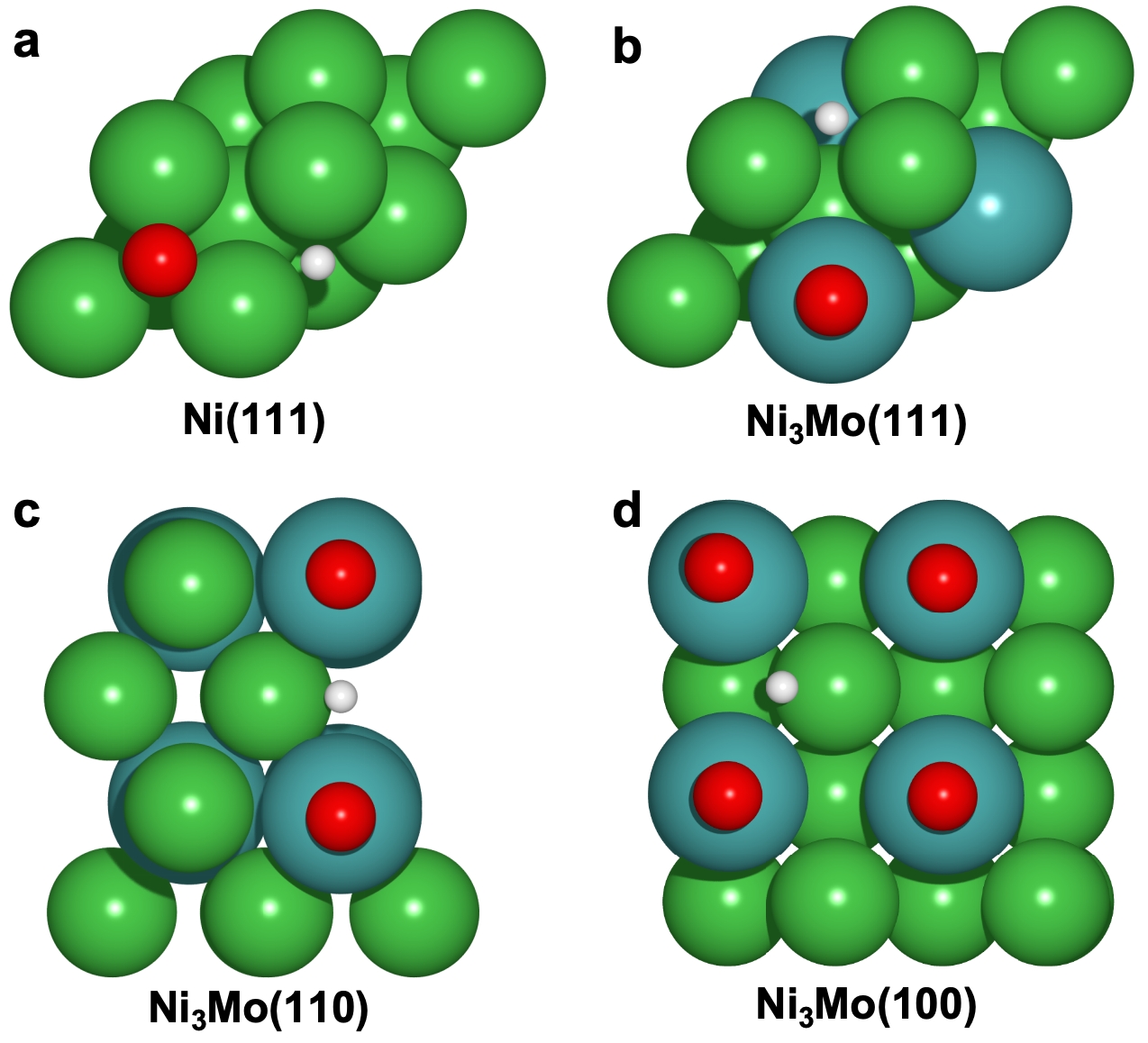


**Figure S2**. The optimized structures of the most active H* intermediate on O*-covered (a) Ni(111), (b) Ni_3_Mo(111), (c) Ni_3_Mo(110), and (d) Ni_3_Mo(100), respectively.


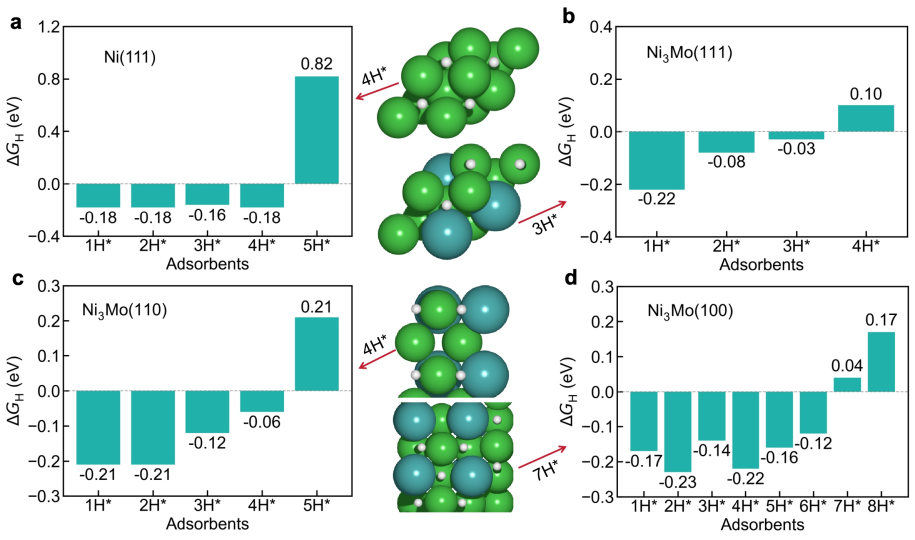


**Figure S3**. Calculated differential hydrogen adsorption free energies (Δ*G*_H_) for (a) Ni(111), (b) Ni_3_Mo(111), (c) Ni_3_Mo(110), and (d) Ni_3_Mo(100), respectively. The middle column shows top views of the most active structures.


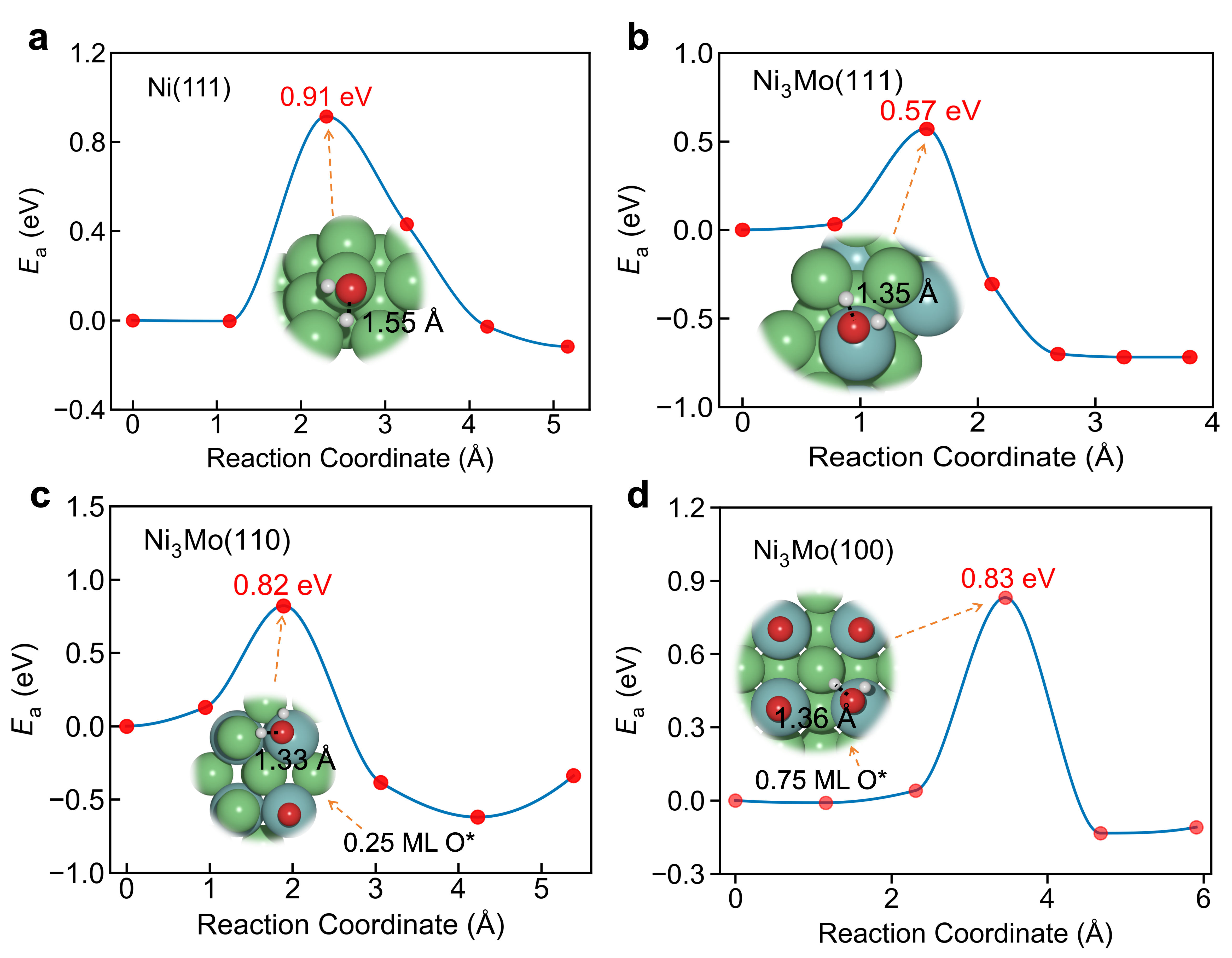


**Figure S4**. Calculated kinetic energy profiles for water dissociation in alkaline conditions on (a) Ni(111), (b) Ni_3_Mo(111), (c) Ni_3_Mo(110), and (d) Ni_3_Mo(100). The inset in each panel shows the corresponding transition state structure.


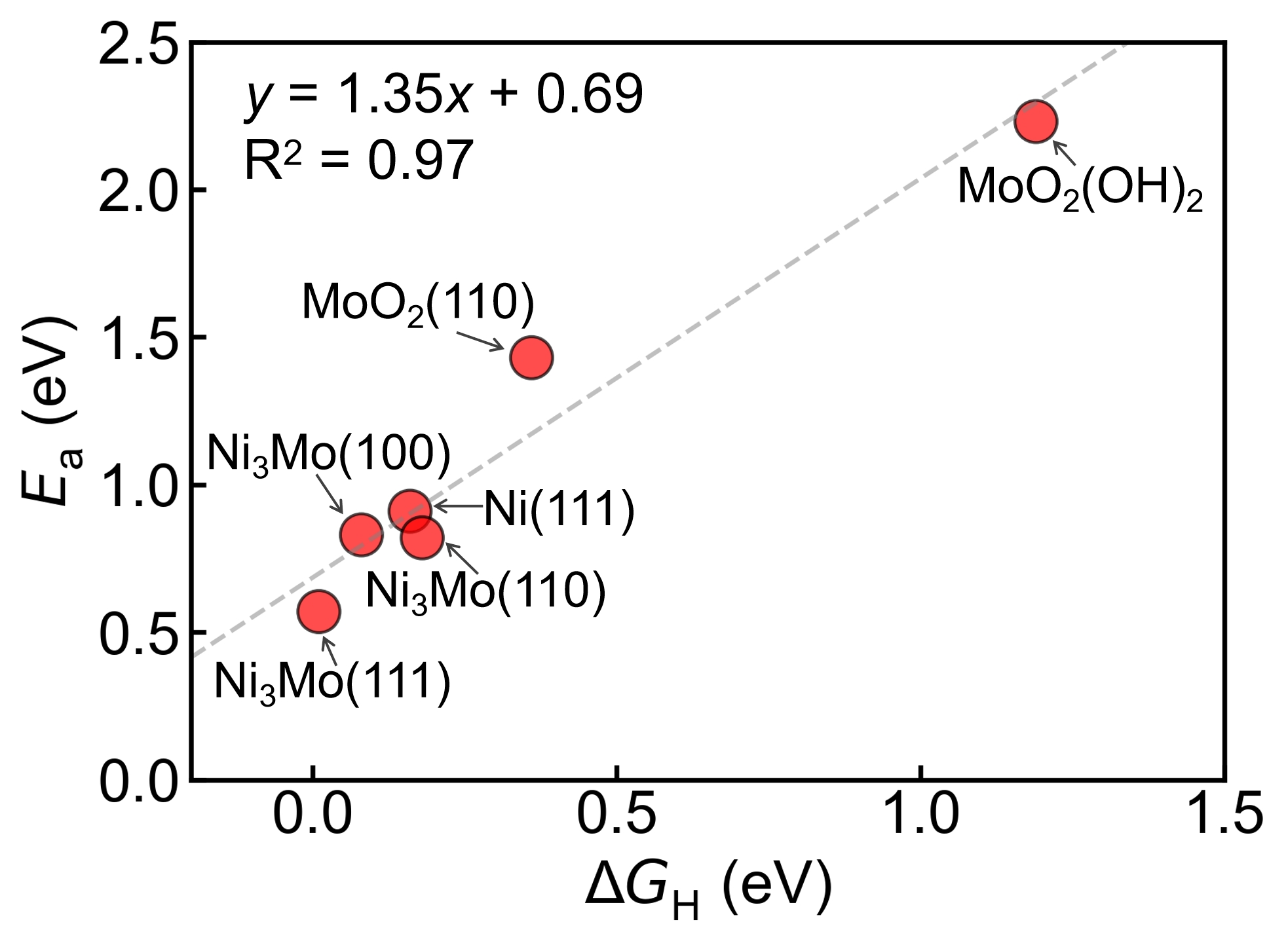


**Figure S5**. The linear scaling between Δ*G*_H_ and *E*_a_ on different catalyst surfaces.


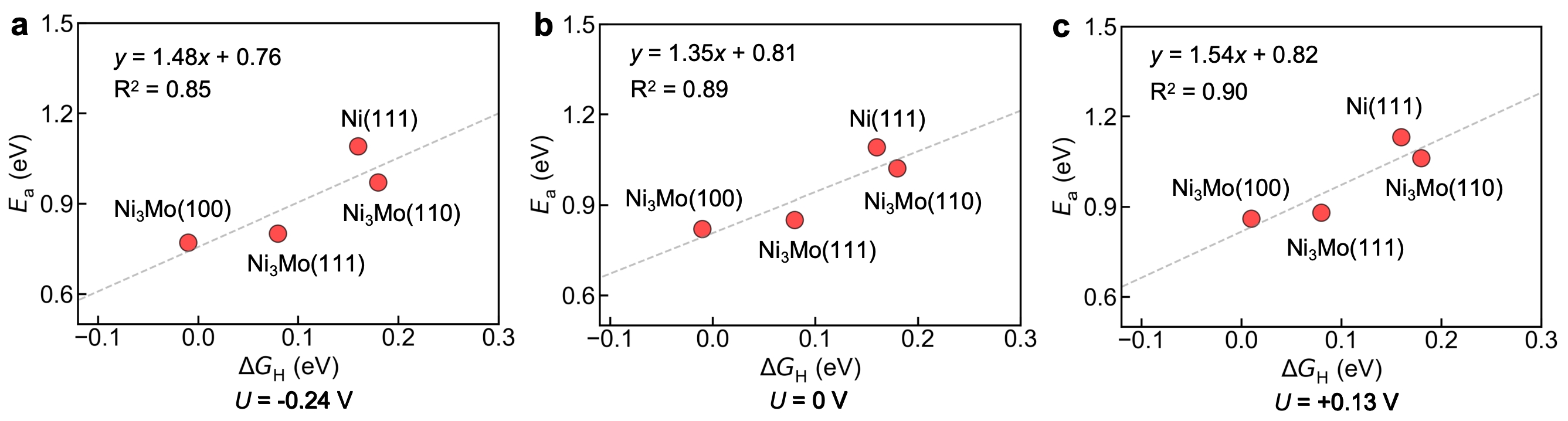


**Figure S6**. The linear scaling between Δ*G*_H_ and *E*_a_ under different applied potentials.


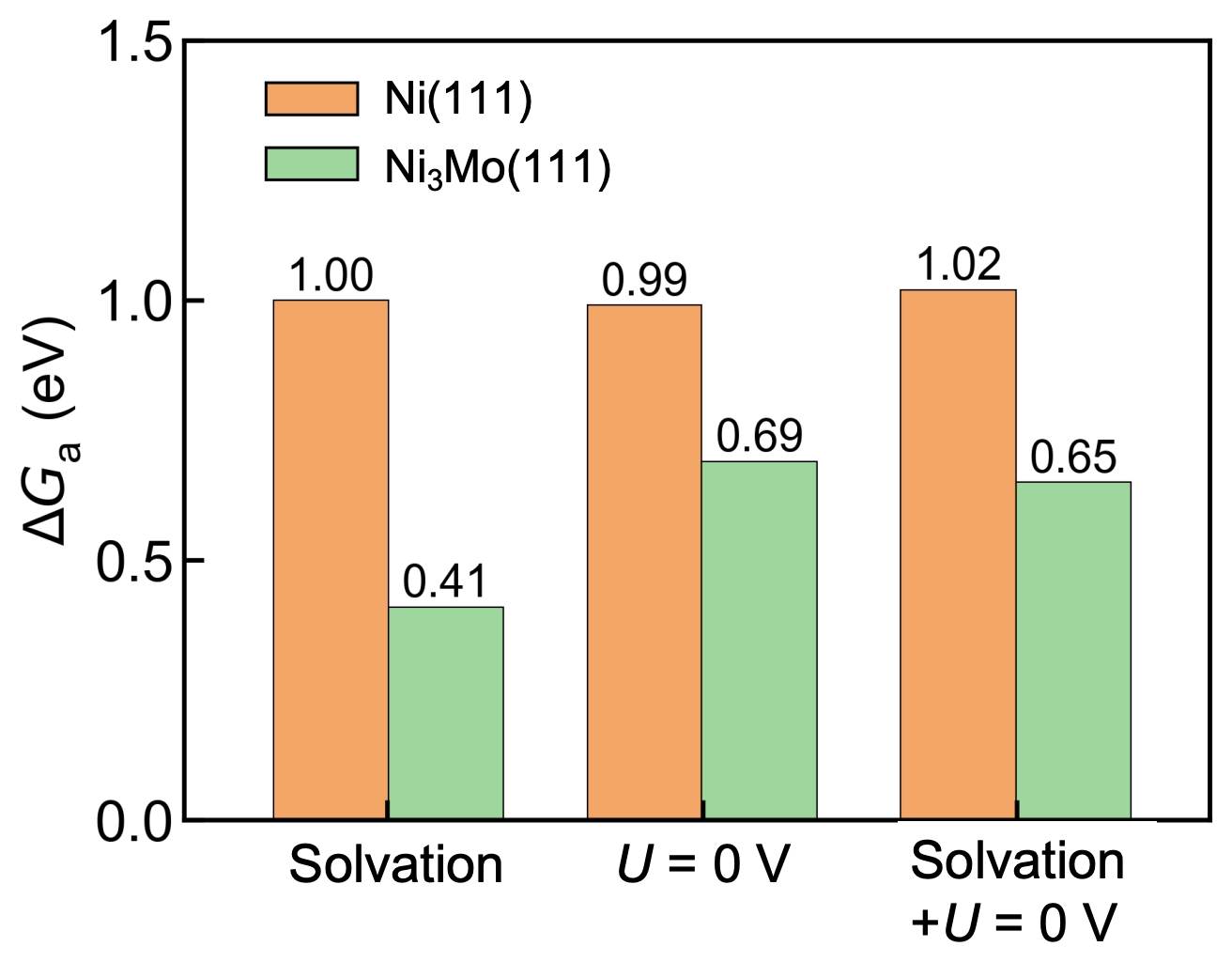


**Figure S7**. Calculated activation free energy (*G*_a_) for water dissociation on Ni(111) and Ni_3_Mo(111) under three conditions: solvation, constant potential (0 V vs. SHE), and both effects combined.


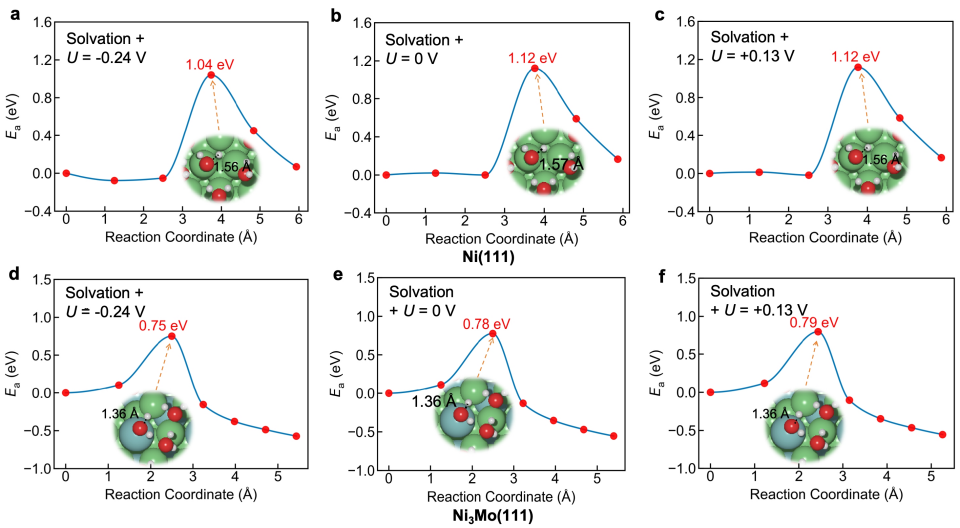


**Figure S8**. Calculated kinetic energy profiles for water dissociation on (a-c) Ni(111) and (d-f) Ni_3_Mo(111) with solvation (modeled explicitly and implicitly) and varying potentials of (a,d) −0.24 V, (b,e) 0 V, and (c,f) +0.13 V versus the standard hydrogen electrode (SHE; 0 V vs. SHE corresponds to −4.44 V on the absolute scale).

Figure S8 presents the calculated water dissociation barriers (*E*_a_) on Ni(111) and Ni_3_Mo(111) surfaces at constant potentials of +0.13, 0.00, and −0.24 V. On Ni(111), *E*_a_ is 1.12, 1.12, and 1.04 eV as the potential is stepped to more negative values. On Ni_3_Mo(111), *E*_a_ decreases from 0.79 to 0.78 to 0.75 eV as the potential becomes more negative. These results are consistent with experimental observations that increasing overpotential increases HER activity/current density for both Ni and NiMo alloys catalysts in alkaline media. Moreover, the *E*_a_ on Ni_3_Mo(111) are always lower than that Ni(111), suggesting fast kinetics for HER activity, which is in excellent agreement with experiments.^[13]^


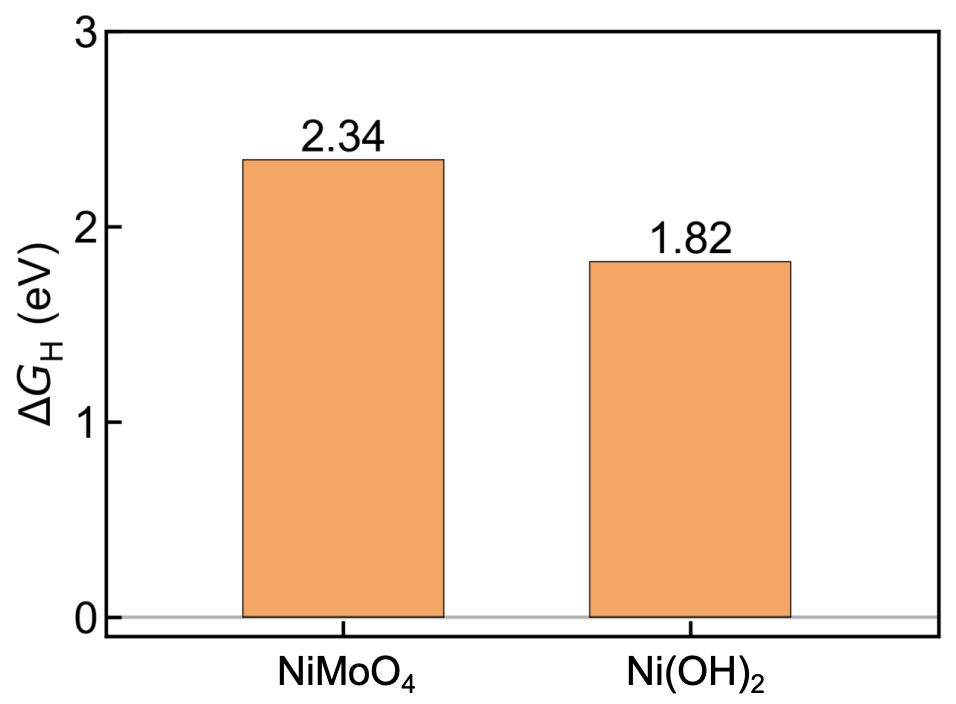


**Figure S9**. Calculated hydrogen adsorption free energies ($\Delta G_{H}$) on NiMoO_4_ and Ni(OH)_2_.


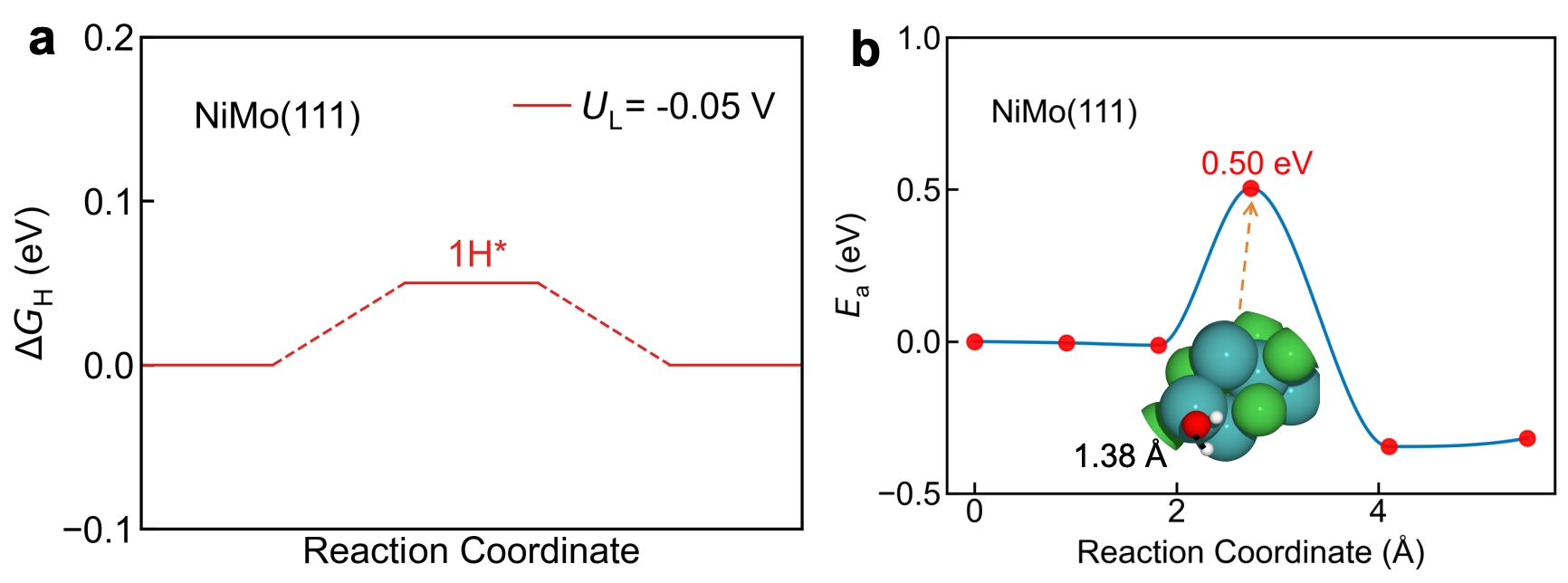


**Figure S10**. Calculated (a) hydrogen adsorption free energies (Δ*G*_H_) and (b) activation barriers (*E*_a_) for water dissociation on NiMo(111).

**Reference:**

[1] J. Hafner, Ab-initio simulations of materials using VASP: Density-functional theory and beyond, *J Comput Chem* **2008**, *29*, 2044–2078;

[2] P. E. Blochl, Projector augmented-wave method, *Phys Rev B Condens Matter* **1994**, *50*, 17953–17979.

[3] L. B. H. a. J. K. N. B. Hammer, Improved adsorption energetics within density-functional theory using revised Perdew-Burke-Ernzerhof functionals, *Phys. Rev. B* **1999**, *59*, 11.

[4] B. P. U. Graeme Henkelman, Hannes Jo´nsson, A climbing image nudged elastic band method for finding saddle points and minimum energy paths, *Journal of Chemical Physics* **2000**, *113*, 9901.

[5] M. Schalenbach, F. D. Speck, M. Ledendecker, O. Kasian, D. Goehl, A. M. Mingers, B. Breitbach, H. Springer, S. Cherevko, K. J. J. Mayrhofer, Nickel-molybdenum alloy catalysts for the hydrogen evolution reaction: Activity and stability revised, *Electrochimica Acta* **2018**, *259*, 1154–1161.

[6] R. B. Patil, A. Mantri, S. D. House, J. C. Yang, J. R. McKone, Enhancing the Performance of Ni-Mo Alkaline Hydrogen Evolution Electrocatalysts with Carbon Supports, *ACS Applied Energy Materials* **2019**, *2*, 2524–2533.

[7] J. J. M. Ask Hjorth Larsen, Jakob Blomqvist et al., The atomic simulation environment—a Python library for working with atoms, *Journal of Physics: Condensed Matter* **2017**, *29*, 273002.

[8] A. H. L. Jens Jørgen Mortensen, Mikael Kuisma et al., GPAW: An open Python package for electronic-structure calculations, *J. Chem. Phys.* **2024**, *160*, 092503.

[9] S. M. R. Islam, F. Khezeli, S. Ringe, C. Plaisance, An implicit electrolyte model for plane wave density functional theory exhibiting nonlinear response and a nonlocal cavity definition, *The Journal of Chemical Physics* **2023**, *159*.

[10] W Tang, E Sanville and G Henkelman, A grid-based Bader analysis algorithm without lattice bias. J. Phys.: Condens. Matter **2009**, 21, 084204.

[11] O. Vinogradova, D. Krishnamurthy, V. Pande, V. Viswanathan, Quantifying Confidence in DFT-Predicted Surface Pourbaix Diagrams of Transition-Metal Electrode-Electrolyte Interfaces, *Langmuir* **2018**, *34*, 12259-12269;

[12] J. R. a. J. K. N. Heine A. Hansen, Surface Pourbaix diagrams and oxygen reduction activity of Pt, Ag and Ni(111) surfaces studied by DFT, *Phys Chem Chem Phys* **2008**, *10*, 3722–3730.

[13] J. R. McKone, B. F. Sadtler, C. A. Werlang, N. S. Lewis, H. B. Gray, Ni–Mo Nanopowders for Efficient Electrochemical Hydrogen Evolution, *ACS Catalysis* **2013**, *3*, 166–169.
